# Developing a platform system for gene delivery: amplifying virus-like particles (AVLP) as an influenza vaccine

**DOI:** 10.1038/s41541-017-0031-7

**Published:** 2017-11-20

**Authors:** Huiling Wei, Zhenhai Chen, Andrew Elson, Zhuo Li, Mathew Abraham, Shannon Phan, Sateesh Kristhnamurthy, Paul B. McCray, Seth Andrews, Steve Stice, Kaori Sakamoto, Cheryl Jones, S. Mark Tompkins, Biao He

**Affiliations:** 1 Department of Infectious Diseases, College of Veterinary Medicine, University of Georgia, Athens, GA 30602 USA; 2 Department of Pediatrics, Pappajohn Biomedical Institute, University of Iowa, Iowa City, IA 52242 USA; 3 Rhodes Center for Animal and Dairy Science, The University of Georgia, Athens, GA 30602 USA; 4 Department of Pathology, College of Veterinary Medicine, The University of Georgia, Athens, GA 30602 USA; 5 Department of Veterinary Preventive Medicine, College of Veterinary Medicine, 225009 Yangzhou, China; 6 University of Alabama at Birmingham, School of Medicine, Birmingham, AL 35294 USA

## Abstract

Delivery of a gene of interest to target cells is highly desirable for translational medicine, such as gene therapy, regenerative medicine, vaccine development, and studies of gene function. Parainfluenza virus 5 (PIV5), a paramyxovirus with a negative-sense RNA genome, normally infects cells without causing obvious cytopathic effect, and it can infect many cell types. To exploit these features of PIV5, we established a system generating self-amplifying, virus-like particles (AVLP). Using enhanced green fluorescent protein (EGFP) as a reporter, AVLP encoding EGFP (AVLP–EGFP) successfully delivered and expressed the EGFP gene in primary human cells, including stem cells, airway epithelial cells, monocytes, and T cells. To demonstrate the application of this system for vaccine development, we generated AVLPs to express the HA and M1 antigens from the influenza A virus strain H5N1 (AVLP–H5 and AVLP–M1H5). Immunization of mice with AVLP–H5 and AVLP–M1H5 generated robust antibody and cellular immune responses. Vaccination with a single dose of AVLP–H5 and M1H5 completely protected mice against lethal H5N1 challenge, suggesting that the AVLP-based system is a promising platform for delivery of desirable genes.

## Virotherapy: using virus-like particles as a ‘trojan horse’

An ‘imitation virus’ can be used to deliver genetic material to target cells, with farreaching potential for medical application. The capacity to safely and affordably introduce genes into cells is highly-sought. A team led by the University of Georgia’s Biao He created a protein shell using parainfluenza virus 5 proteins, with the resultant particles possessing the ability to infect multiple types of cell and deliver desired genetic material. The team proved the utility of their system by using it to express immunity-promoting components of avian influenza virus in live mice—successfully vaccinating the animals, and enabling them to survive a subsequent lethal infection. His group also showed that their system is also able to deliver and express genes in human cells, prompting further research into this useful tool.

## Introduction

Advances in understanding diseases have created great opportunities to prevent, treat, and manage them. For instance, mutations in cystic fibrosis transmembrane conductance regulator (*CFTR*) gene have been identified in people with cystic fibrosis. However, the delivery of genes of interest to target cells remains a critical hurdle for translating the potential of new knowledge. Many strategies, based on viral and non-viral vector systems, have been explored for gene delivery. At present, all of these strategies have shortcomings. It is highly desirable to have a gene delivery system that is safe, efficacious, versatile, and easy to produce.

PIV5, a non-segmented, negative-stranded, RNA virus (NNSV), is a member of the *Rubulavirus* genus of the family *Paramyxoviridae*, which includes mumps virus (MuV).^1^ PIV5 encodes eight known viral proteins.^1^ The nucleocapsid protein (NP), phosphoprotein (P), and large RNA polymerase (L) protein are important for transcription and replication of the viral RNA genome. NP encapsidates the viral RNA genome into the ribonucleocapsid (RNP), and this RNP is the functional template for RNA synthesis. The V/P gene encodes two mRNA species that translate into the V and the P proteins through a non-templated insertion of two G residues at a specific site of the V/P gene during transcription.^2^ The V protein plays important roles in viral pathogenesis, as well as in regulating viral RNA synthesis.^1,3^ The fusion (F) protein, a glycoprotein, mediates both cell-to-cell and virus-to-cell fusion in a pH-independent manner. The hemagglutinin-neuraminidase (HN), another viral glycoprotein, is also involved in virus entry and release from the host cells. The matrix (M) protein plays an important role in virus assembly and budding.^4,5^ PIV5 virus-like particles (VLP) can be formed with HN or F plus M.^6^ However, the addition of NP enhances VLP formation. The small hydrophobic (SH) protein is a 44-residue hydrophobic integral membrane protein and is oriented in membranes with its N terminus in the cytoplasm.^7^

One unique feature of PIV5 is its ability to infect virtually any mammalian cell without causing cytopathic effect in many of them, allowing the virus to grow for long periods of time in infected cells. It is known that many cells are persistently infected with PIV5.^8^ The broad tropism of PIV5 is likely due to its HN protein that binds to sialic acid, a ubiquitous modification of cell surface proteins, which activates F to fuse the PIV5 virion and host cell plasma membrane, resulting in entry of the PIV5 genome.

Influenza A virus (IAV) VLPs containing HA, NA, and M1 have been developed as potential vaccines.^9–11^ They are effective in generating robust humoral and cellular immune responses^12^ and have been shown to be effective in human clinical trials.^13^ A new generation of influenza VLP containing M2 of IAV has also been developed.^14^ One challenge for using influenza VLPs as a vaccine is the production of the large amounts of VLP needed for immunization.

In this work, we have generated PIV5 VLPs that can deliver and amplify foreign genes in target cells without producing progeny (Amplifying Virus-Like Particle, AVLP). To demonstrate the applications of the system, we used AVLPs to express antigens of IAV and tested their efficacy in an animal model.

## Results

### Generation of AVLP expressing enhanced green fluorescent protein (EGFP)

**Fig. 1:**
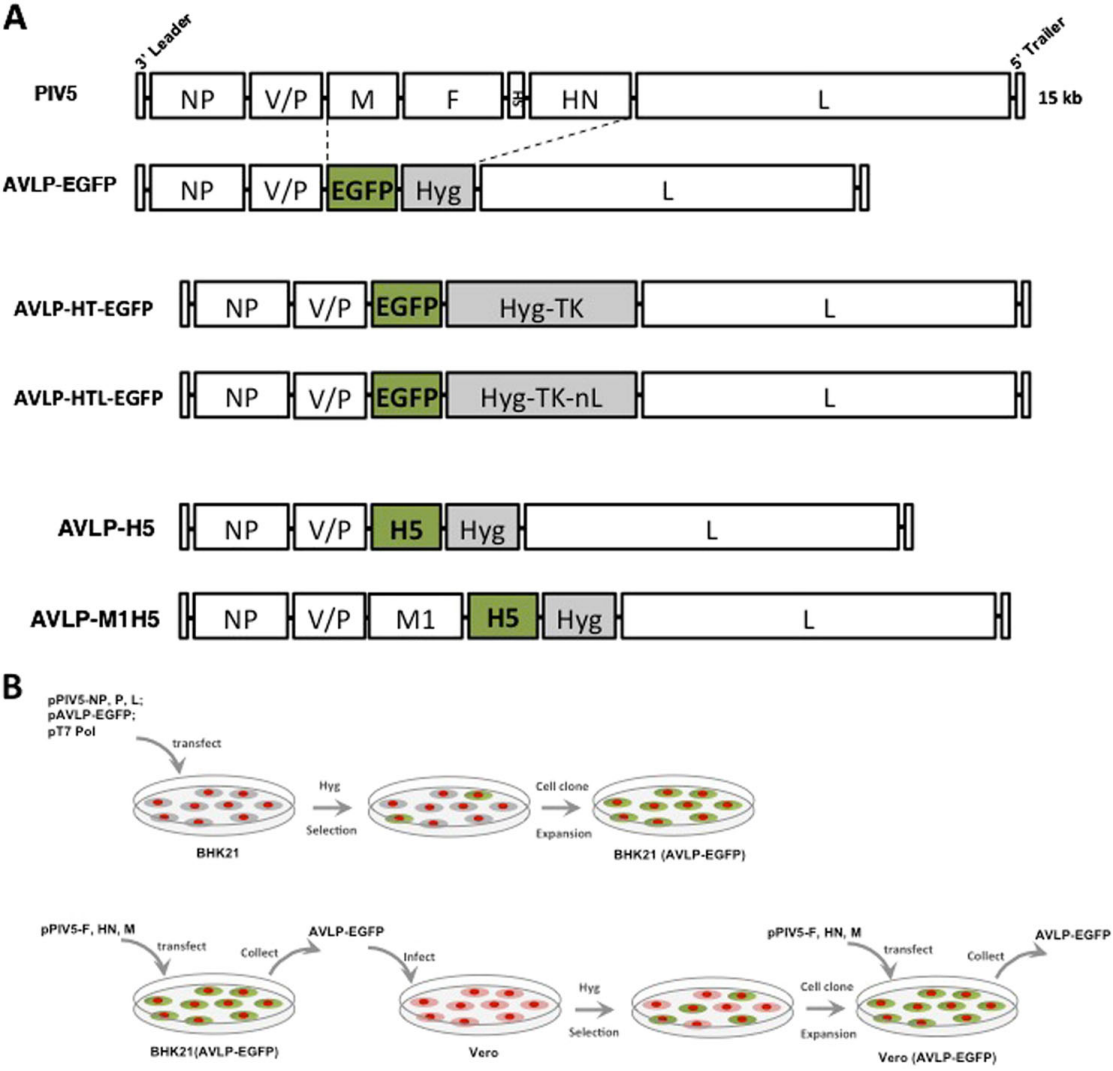
Generation of PIV5 AVLP constructs. **a** Schematics of PIV5, AVLP–EGFP, AVLP–H5, AVLP–M1H5, AVLP–HT–EGFP, and AVLP–HTL–EGFP. Leader and trailer sequences are important for PIV5 RNA synthesis. NP nucleoprotein, P phosphoprotein, V V protein, M matrix protein, SH small hydrophobic protein, F fusion protein, HN Hemagglutinin-neuraminidase protein, L RNA-dependent RNA polymerase, Hyg hygromycin, EGFP enhanced green fluorescent protein, H5 HA of H5N1, M1 M1 of H5N1, TK thymidine kinase, nL nano-luciferase. **b** Schematics of AVLP generation work flow. BHK21 cells were transfected with plasmids expressing PIV5 P, NP, L, and T7 polymerase together with PIV5 AVLP plasmid expressing EGFP. The transfected cells were then selected with hygromycin. BHK (AVLP–EGFP) cell clones containing PIV5 AVLP genome were expanded under the selection of Hygromycin. BHK(AVLP-EGFP) cells were transfected with plasmids expressing PIV5 F, HN, and M. Particles carrying the PIV5 AVLP genome packaged by PIV5 HN, F, and M were generated in the supernatants of transfected cells. The particles were designated as AVLP–EGFP and used to infect Vero cells. The infected Vero cells were developed into stable cell lines carrying PIV5 AVLP genome [Vero (AVLP–EGFP)] under selection of Hygromycin. Vero (AVLP–EGFP) cells were transfected with plasmids expressing PIV5 F, HN, and M, and then AVLP–EGFP particles were produced in the supernatants of transfected cells. H.W. created this figure

We constructed a plasmid containing the PIV5 genes NP, V/P, and L that encode the NP, P, and L, respectively, with regulatory sequences (leader, trailer, and appropriate gene junctions sequences) (Fig. 1a). We inserted the EGFP gene downstream of the V/P gene as a reporter for easy tracking in live cells. To allow selection of cells containing AVLP, we inserted a selectable marker, hygromycin B resistance gene (Hyg), in the AVLP (Fig. 1a). The plasmid pAVLP-EGFP, along with plasmids pCAGGS-PIV5-L, pCAGGS-PIV5-NP, pCAGGS-PIV5-P, and pBH437 (expressing T7 RNA polymerase), were transfected into BHK21 cells. The transfected cells were passaged and selected in hygromycin for 2–3 weeks. Individual colonies of selected cells were expanded (Fig. 1b). Green fluorescence was observed in selected BHK21 cells (Fig. S1A).

**Fig. 2:**
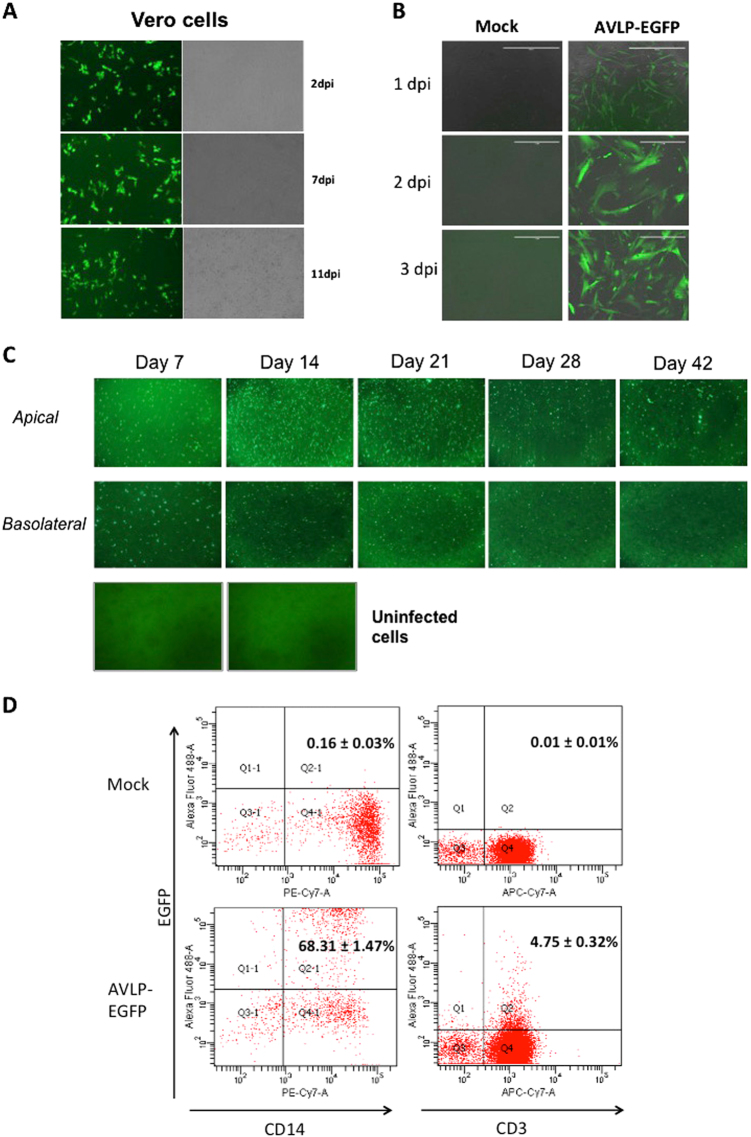
Infection of AVLP–EGFP to primary and continuous cell lines. **a** EGFP expression in Vero cells. **b** EGFP expression in human MSC cells. **c** EGFP expression in human primary epithelial cells. **d**. EGFP expression in human PBMCs. H.W. created this figure

F, HN, and M play important roles in PIV5 assembly and budding.^5^ To obtain particles from the cells containing the AVLP–EGFP genome, the cells were transfected with the plasmids expressing PIV5 F, HN, and M (Fig. 1b), and supernatants containing single-cycle, infectious, PIV5 particles (AVLP–EGFP) were filtered through a 0.2 µm filter. The clarified supernatants from the transfected cells were used to “infect” fresh cells. As shown in Fig. 2a, expression of EGFP was observed in Vero cells up to 11 days post-inoculation, indicating that infectious AVLP–EGFP was produced. Expression of EGFP was also detected in HeLa cells (Fig. S1C). AVLP–EGFP infected Vero cells were selected with Hyg and expanded as a stable cell line to produce AVLPs, Vero (AVLP–EGFP) (Fig. S1A). The expression of PIV5 proteins was detected by immunoblotting with antibodies specific for PIV5 NP and P (Fig. S1B). Furthermore, we purified RNAs from the cells and sequenced AVLP genomes from these cells using a RT-PCR product. The AVLP RNA sequences were identical to the input cDNA sequence in the plasmid pAVLP–EGFP. We determined the number of infectious particles in media by counting numbers of cells showing GFP at 1 day post-inoculation (DPI). AVLP titers were greater than 10^6^ infectious particles per milliliter (AP/ml). When we applied the media from AVLP–EGFP-infected cells to fresh cells, no EGFP was observed, indicating that no infectious progeny was produced when HN, F, and M were not present.

The ability of AVLP–EGFP to express EGFP in primary cells was examined in human mesenchymal stem cells (MSC) (Fig. 2b) and in primary cultures of well-differentiated human airway epithelial cells (Fig. 2c). EGFP was observed in these cells for as long as 42 DPI. Furthermore, in human peripheral blood mononuclear cells (PBMCs), CD3^+^ and CD14^+^ cells expressed EGFP (Fig. 2d), indicating that AVLP–EGFP successfully infected and expressed EGFP in T cells and monocytes. In addition, EGFP expression was observed in canine MSC and primary porcine airway epithelial cells (Fig. S1D and S1E). These results indicate that AVLP–EGFP is a versatile system that allows the expression of genes of interest in many primary cells and immortalized cell lines.

### Generation of an AVLP with a self-destructive gene and a reporter

**Fig. 3:**
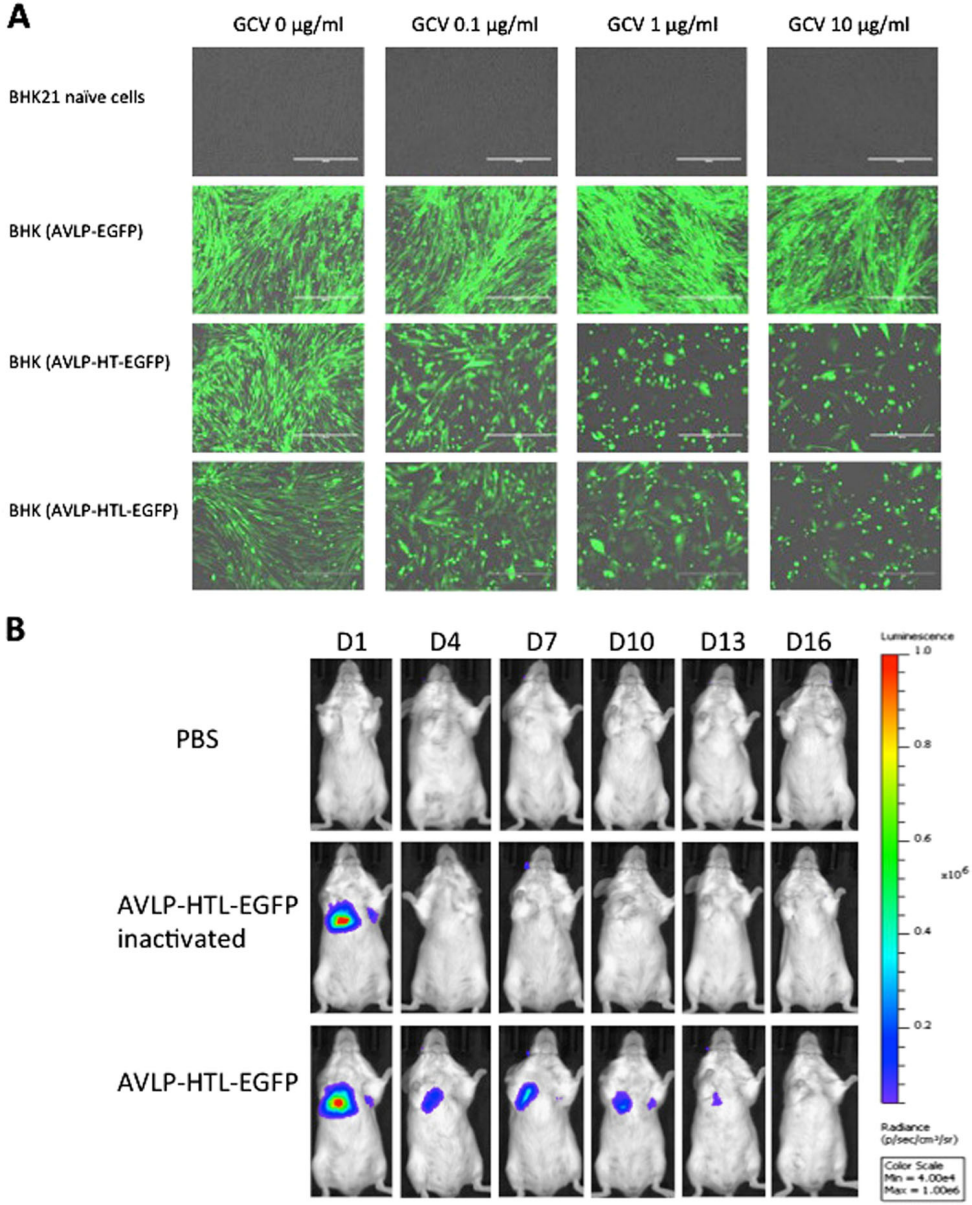
Examination of killing efficiency by GCV in established stable cell lines and monitoring of the expression kinetics of AVLP–HTL–EGFP in vivo. **a** Effects of GCV on AVLP-containing cells. BHK21 stable cell line containing AVLP–, AVLP–HT–, or AVLP–HTL–EGFP were treated with GCV at various concentrations. The images were taken at 3 days postinfection using a fluorescence microscope. **b** Detection of AVLP in vivo. BALB/c mice were infected with AVLP–HTL–EGFP and monitored for expression of nL using an IVIS camera. H.W. created this figure

Even though AVLP–EGFP cannot produce progenies, we have nonetheless added a safety feature. It is known that cells with a functional thymidine kinase (TK) gene product can be killed in the presence of ganciclovir (GCV), a FDA-approved drug. We fused the TK gene with Hyg (HT) to generate AVLP–HT–EGFP (Fig. 1a). In addition, we added a nano-luciferase (nL) gene to the HT gene to generate AVLP–HTL–EGFP (Fig. 1a) and allow tracking of AVLP in vivo. Expression of Hyg, Hyg–TK fusion, and Hyg–TK–nL fusion in the cell lines that contain AVLP–HT–EGFP and AVLP–HTL–EGFP were confirmed by immunoblotting (Fig. S2A). GCV killed cells containing AVLP–HT–EGFP and AVLP–HTL–EGFP in a dose-dependent manner, while it had no effect on cells containing AVLP–EGFP (Fig. 3a and Fig. S2B).

To track AVLP in vivo, we inoculated BALB/c mice with AVLP–HTL–EGFP, and luciferase activities were detected for 16 days after infection using an IVIS camera (Fig. 3B and S2C).

### Generation of AVLP expressing HA of H5N1 (AVLP–H5)

**Fig. 4:**
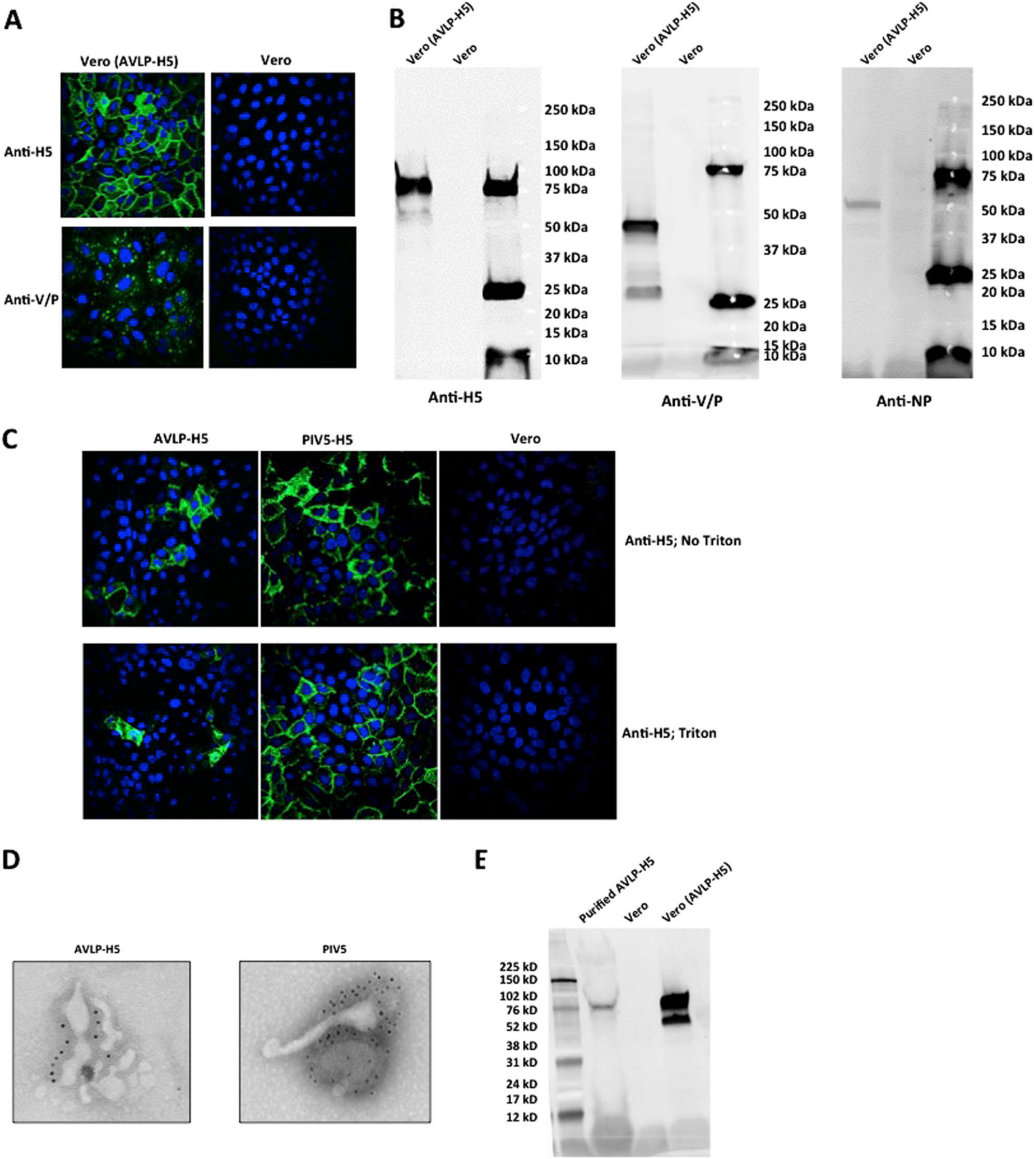
Characterization of Vero (AVLP–H5) cell line and Vero cells infected with AVLP–H5. **a** Detection of H5 and PIV5 V/P expression in Vero (AVLP–H5) cells by IFA. The cells were fixed and stained with anti-H5 or anti-PIV5 V/P antibodies followed by staining with FITC- conjugated secondary antibody. DAPI staining was performed with ProLong® Gold Antifade Mountant. **b** Detection of H5, PIV5 V/P, and NP expression in cells by western blotting. The Vero (AVLP–H5) cell samples were stained with anti-H5, anti-PIV5 V/P, or anti-PIV5 NP antibodies. Vero cell samples were used as a negative control. Samples derived from the same experiment and gels/blots were processed in parallel. **c** Surface expression of H5. Two days after infection with AVLP–H5 or PIV5–H5, Vero cells were fixed and treated with triton or PBS. The cells were stained with anti-H5 antibody and ProLong^®^ Gold Antifade Mountant as in **a**. **d** Analysis of particles. The concentrated AVLP-H5 and PIV5 particles were treated with anti-PIV5 HN antibody and then secondary antibody labeled with gold particles. The samples were examined using an electron microscope. **e** H5 incorporation. The purified AVLP-H5 particles were subjected to WB analysis with H5 specific antibody. Samples derived from the same experiment and gels/blots were processed in parallel. H.W. created this figure

To test the AVLP system as a potential vaccine platform, we generated AVLP expressing the HA gene of H5N1 (H5)(AVLP–H5) (Fig. 1a) using a HA gene whose polybasic cleavage site was removed.^15^ H5, NP, V, and P expression was detected in AVLP–H5 cells (Figs. 4a, b). Interestingly, a Val to Trp mutation at amino acid residue 5 in the H5 gene was observed in AVLP–H5 in Vero cells. The H5 protein was detected in AVLP–H5 and PIV5–H5 (recombinant PIV5 expressing H5HA protein)^12^ infected Vero cells and was observed to localize mainly to the cell membrane (Fig. 4c), suggesting that this single mutation in the H5 gene of AVLP–H5 did not affect the localization pattern of H5. AVLP–H5 particles were used to infect fresh cells and supernatant from the infected cells was harvested and the supernatant used to infect fresh cells. Protein expression was only detected in the first round of infection (data not shown), indicating that no infectious particles were produced from AVLP–H5 cells without transfecting the cells with PIV5 M, F, and HN.

The AVLP–H5 particles were obtained from supernatants of Vero (AVLP–H5) cells transfected with plasmids encoding PIV5 F, HN, and M at a titer about 4 × 10^6^ AP/mL. For visualization by transmission electron microscopy, the concentrated AVLP–H5 particles were fixed and stained with an anti-PIV5 HN antibody. We observed that the AVLP particles were similar in size and shape to wild-type PIV5 particles (Fig. 4d). While we were able to detect H5 in sucrose gradient-purified AVLP–H5 particles using WB (Fig. 4e), we did not detect H5 using EM. It might indicate that a very limited number of HA molecules were packaged into AVLP particles, that cell debris containing HA was co-purified with AVLP particles, or the HA band in WB might be from HA aggregates, which had a similar density as AVLPs in gradient centrifugation.

### Immune responses of AVLP–H5 and efficacy against lethal H5N1 challenge in mice

To investigate whether AVLPs could elicit HA-specific antibodies in vivo, mice were vaccinated intranasally with PBS, PIV5–H5, or AVLP–H5. For AVLP–H5, a boost was performed 19 days after the initial immunization. Two doses of AVLP–H5 or one dose of PIV5–H5 vaccination induced specific anti-H5–HA antibodies (Fig. S3A). Six of eight AVLP–H5-immunized mice exhibited detectable hemagglutination inhibition (HAI) titers between 10 to 40, while six of seven PIV5–H5-vaccinated mice had HAI titers within the same range (Fig. S3B). Overall, the ELISA and HAI assay results suggest that similar humoral immune responses against H5 were elicited in the PIV5–H5 and AVLP–H5 vaccinated mice.

The immunized mice were challenged with 10 LD_50_ of H5N1 at 42 days after the initial immunization. All control PBS-immunized mice lost body weight and succumbed to the infection. In contrast, 100% of mice vaccinated with PIV5–H5 (10/10 mice) or AVLP–H5 (15/15 mice) survived this lethal influenza challenge with no significant weight loss (Fig. S3C and Fig. S3D). These results indicate that two doses of the AVLP–H5 vaccine provided robust protection comparable to the live PIV5–H5 vaccine.

**Fig. 5:**
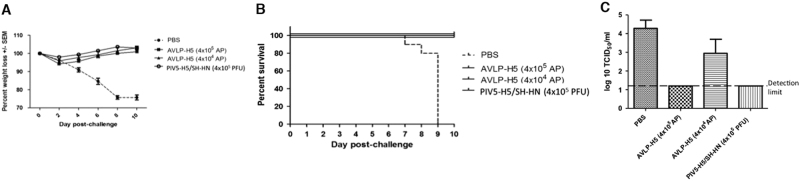
A single dose immunization of AVLP-H5 protects against H5N1 virus challenge. Mice in a group of 15 were vaccinated with a single dose of PBS, PIV5–H5 4 × 10^5^ PFU per mouse), or AVLP–H5 intranasally at 4 × 10^4^ or 4 × 10^5^ AP. At 25 days post vaccination, mice were challenged with 10 LD_50_ of H5N1. Weight loss **a** and survival **b** were monitored for 10 days following challenge. Weight loss is graphed as an average percentage of the original weight (the day of challenge; ± SEM). On day 4 post-challenge, a subset of animals was humanely euthanized, and tissues were collected for lung virus titers **c**. The limit of detection in **c** was 1.2 log_10_ TCID_50_/ml. S.M.T. created this figure

We next immunized mice with a single dose of AVLP–H5 at 4 × 10^4^ or 4 × 10^5^ AP per mouse. At 21 days after the immunization, mice were euthanized, and splenocytes were obtained for IFN-γ ELISPOT assays. Compared to PBS control mice, the PIV5–H5 and AVLP–H5 vaccinations induced specific and similar levels of H5-specific cellular immune responses (Fig. S3E). We also measured neutralizing antibody against PIV5 and found that PIV5–H5 generated measurable anti-PIV5 neutralizing antibody. AVLP–H5 immunizations (single dose or prime-boost) did not generate any measurable anti-PIV5 neutralizing antibody (Fig. S3F). Excitingly, both single dose immunizations completely protected mice from death (Figs. 5a, b). At the 4 × 10^5^ AP dose, no H5N1 was detected in the lungs of immunized mice 4 days after challenge, indicating that a single dose of AVLP-H5 was sufficient to provide sterilizing immunity against lethal H5N1 challenge in mice (Fig. 5c).

### Generating AVLP expressing HA and M1 of H5N1 (AVLP–M1H5)

**Fig. 6:**
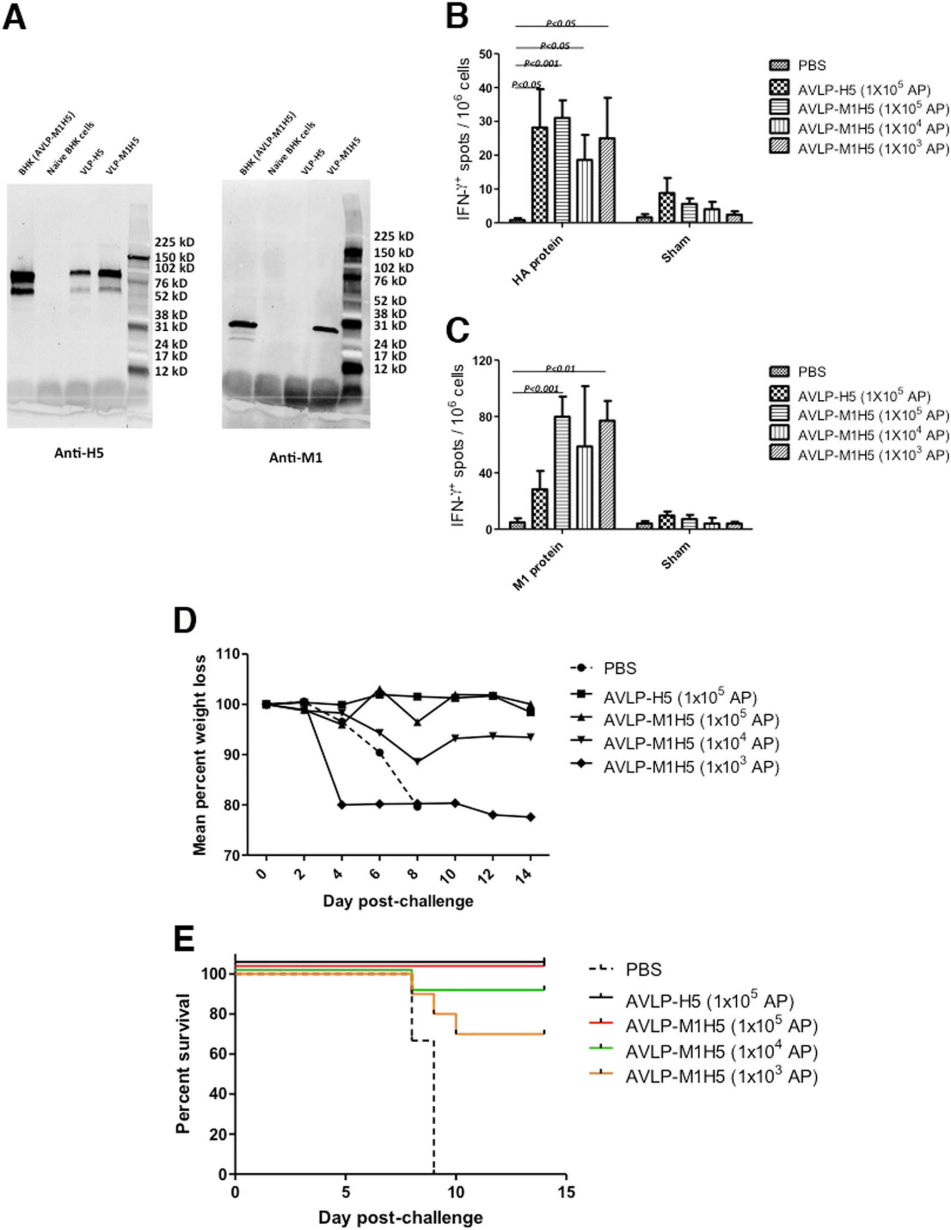
A single dose immunization of AVLP–M1H5 protects against H5N1 virus challenge. **a** Detection of M1 and H5 in cell media. Supernatants from BHK (AVLP–H5) and BHK (AVLP–M1H5) cell cultures were purified and subjected to WB. Samples derived from the same experiment and gels/blots were processed in parallel. **b** and **c** ELISPOT assay for IFN-γ. Mice in a group of 5 were vaccinated with a single dose of PBS, AVLP–H5 (1 × 10^5^ AP), or AVLP–M1H5 intranasally (1 × 10^5^, 1 × 10^4^ or 1 × 10^3^ AP). At day 21 post-prime immunization, mice were sacrificed, and spleens were collected. Splenocytes were stimulated with PBS, H5, or M1 recombinant protein. Results are presented as the mean number of IFN-γ-producing cells per 10^6^ splenocytes. Differences were evaluated by one-way ANOVA. **d** and **e** morbidity and mortality after challenge. H.W. created figures **a** and **b**. S.M.T. created figures **c** and **d**

It is known that the HA and M1 of IAV can form VLP.^16,17^ VLP containing M1 and HA has been explored as an IAV vaccine.^10,17,18^ We, therefore, generated AVLP expressing both H5 and M1 (AVLP–M1H5) (Fig. 1a). As expected, the cells containing AVLP–M1H5 expressed H5 on the cell surface (Fig. S4), and M1 and H5 expression were detected in the cells by WB (Fig. 6a). Interestingly, M1 and H5 were also detected by WB in the sucrose gradient-purified particles from BHK (AVLP–M1H5) cell culture supernatant (Fig. 6a), indicating that M1 and H5 formed influenza-like VLPs (VLP–M1H5) that were released into the media. A band corresponding to HA was detected in the supernatant from BHK (AVLP–H5) by WB at a lower level than AVLP–M1H5. It is possible that H5HA by itself could be released randomly into the cell culture medium inefficiently, cell debris containing HA were co-purified with AVLPH5, or that HA just aggregated into “rosettes” due to interactions of the hydrophobic domains that happened to have a similar density as VLP in gradient centrifugation. To determine the immunogenicity and efficacy of AVLP–M1H5 as a vaccine, mice were immunized with different doses of AVLP–M1H5 (10^3^ to 10^5^ AP per mouse) intranasally. All doses of AVLP–M1H5 produced cellular immune responses similar to 10^5^ AP of AVLP–H5 (Fig. 6b), and AVLP–M1H5 also induced M1-specific cellular immune responses (Fig. 6c). After lethal H5N1 challenge, we observed some weight loss (Fig. 6d). The single dose immunization with 10^3^ AP protected 70% of mice, the 10^4^ dose protected 90% of mice, and the 10^5^ dose protected 100% of mice (Fig. 6e).

## Discussion

PIV5 can infect a diverse variety of primary human and animal cells.^19^ The ability of PIV5-based AVLP to infect many mammalian cell types allows this system to be used as a gene delivery platform for primary cells. Since the AVLP genome is negative-sense RNA, it is unlikely to integrate its genome into host chromosomes. In the absence of selective pressure, cells will likely gradually lose the AVLP genome. Because of the transient nature of PIV5-based AVLP, this system is well suited to deliver genes and/or RNA sequences into primary cells where a limited duration of expression is preferred. For instance, delivering transgenes into stem cells to drive differentiation or transiently expressing CRISPR/Cas9 genes for genome editing. Expression of EGFP in AVLP–EGFP-containing human primary epithelial cells lasted over a period of 42 days without obvious sign of cytotoxicity, suggesting that the AVLP system can deliver genes of interest to primary cells and sustain the expression of the genes over a long time period. However, in mice, reporter gene expression from AVLP–HTL lasted for only two weeks. While two-week expression of genes of interest may be sufficient and even preferred for vaccine development, for some applications, such as gene therapy, longer-term expression of therapeutic genes of interest will be desirable. One important difference regarding PIV5 replication in human vs. mouse cells is the ability of PIV5 to evade host interferon (IFN) responses. PIV5 cannot evade IFN action in mouse cells, while it does so in human cells.^20^ Thus, it is anticipated that expression of genes by AVLP will last longer in humans than in mice. In addition, it may be possible to influence the duration of expression of a gene of interest from AVLP by modifying its response to IFN.

Using the F and HN of PIV5 to form AVLP allows them to infect many cell types similar to the natural PIV5, allowing PIV5-based AVLP to target as many cell types as possible after delivery. In addition to PIV5 F and HN, other envelope glycoproteins can be used to generate particles from AVLP-infected cells. The pseudotyping of PIV5 AVLP with other glycoproteins provides the ability to target specific cell types.

H5N1 avian IAV emerged in Southeast Asia and resulted in the destruction of millions of birds, and threatens to become the next pandemic. Currently, the only FDA-approved vaccine against H5N1 has serious limitations, particularly as it has to be given twice and requires substantially higher vaccine concentrations to achieve a moderate level of efficacy compared to conventional influenza vaccines.^21,22^ Conventional vaccines utilizing the HA and NA of H5N1 viruses have been poorly immunogenic and have safety and production issues (reviewed in ref. 23). A live-attenuated H5N1 vaccine has been generated by reverse genetics,^24^ but the risk of generating a reassortment prohibits the use of this vaccine in most instances. Inactivated virus vaccines have also been derived by reverse genetics^25,26^ and produced in large quantities, but preliminary results from NIAID clinical trials suggest that efficacy will require both multiple immunizations and six times the standard IAV antigen dose, i.e., 90 µg instead of 15 µg of antigen, while only providing protection in a subset (~50%) of vaccinated individuals.^21,22^ Nonetheless, the FDA has approved the inactivated H5N1 vaccine for use in people between ages 18 and 64, a population that is not the most vulnerable to IAV infection. Thus, there is a need for new vaccine strategies that provide increased immunogenicity and safety.

In this work, we developed a PIV5-based VLP expressing the HA and M1 of H5N1. Because PIV5 VLP can enter many cells and amplify its genome, AVLP containing HA can also enter many cells and amplify the HA gene, allowing de novo synthesis of HA in cells. When HA is expressed with M1 in the cells that are infected with AVLP expressing both HA and M1, de novo M1–HA particles can form, thus amplifying VLP production in vivo. As a result, the amount of AVLP needed for effective immunization is likely less than inactive VLPs. Furthermore, in this work, a mutant form of HA from H5N1, which was not functional, was used. Thus, the newly synthesized M1–H5 particles are not infectious. However, if a functional HA was used in AVLP–M1H5, newly formed M1–H5 particles would be able to enter cells. If these M1–H5 particles contained the AVLP–M1H5 genome, a new round of expression of M1 and H5 would be possible in the newly infected cells, making AVLP-based system more advantageous than a traditional single-cycle replicon system. In our work, AVLP was produced at a titer over 10^6^ AP/ml, and a single dose of 1 × 10^5^ was efficacious (no mortality, no weight loss, and no challenge virus detected in lungs) in protecting mice against lethal H5N1 challenge, demonstrating that an AVLP-based system is feasible as a vaccine platform, and the dose requirement was reasonable, indicating that it is possible to produce AVLP at a reasonable cost. Furthermore, PIV5 can grow to 10^8^ PFU/ml in cells. Our AVLP–H5 grew to 4 × 10^6^ AP/ml. It is known that cells transfected with F, HN, M, and NP can produce numbers of virus-like particles similar to PIV5 infection.^6^ Thus, it is possible to further optimize our current system to increase AVLP titers.

Previously, when we expressed foreign genes, such as IVA HA (PIV5–HA) and the G protein of rabies virus (PIV5–G), HA and G were introduced into virions of PIV5–HA and PIV5–G,^27^ likely due to random incorporation events. We did not detect H5 in AVLP–H5 by immunostaining using EM but detected H5 in AVLP–H5 particles by immunoblot. We speculate that this was caused by the relatively low level of H5 incorporation into AVLP–H5 particles. If necessary, we can modify H5 to allow more efficient incorporation into AVLP, by replacing the cytoplasmic tail of HA with that of PIV5 F, which is a known packaging signal for PIV5 assembly and budding.^28^

In summary, we have developed a new platform for delivering genes of interest. Using this platform for vaccine development can resolve many challenges facing the VLP-based system. Furthermore, this system can be used to deliver desirable genes and sequences of interest to primary cells, leading to new strategies for cancer therapy and regenerative medicine.

## Methods

### Cells

Tissue culture cells were maintained in Dulbecco’s modified Eagle medium (DMEM) with 10% fetal bovine serum (FBS) and 1% penicillin (Pen)-streptomycin (Strep). Human Mesenchymal Stem Cells (MSCs, Lifeline Cell Technology, Frederick MD, USA) were plated at 5000 cells/cm^2^ on tissue culture flasks in complete media (MEM-α, Gibco), 10% defined fetal bovine serum (Hyclone, Logan UT), 2 mM L-glutamine, and 50 U/mL Pen-Strep, and allowed to grow to 80–90% confluency. Human PBMCs were cultured in RPMI-1640 with 10% FBS, 1x Pen/Strep/glutamine, 1x non-essential amino acids, and 1x sodium pyruvate. Airway epithelial cells from human or porcine trachea were cultured at the air-liquid interface (ALI) as described previously.^29^ Methods were performed in accordance with relevant regulations and guidelines approved by institutional biosafety committee.

### Viruses

PIV5-H5 virus has been described previously (12). To concentrate PIV5 or AVLP–H5 particles, the supernatants was first cleared by centrifugation at 3000 r.p.m. for 10 min. The cleared supernatant was loaded onto 20% sucrose and pelleted in a Thermo Scientific ultracentrifuge Type F40L-8 × 100 rotor at 37,000 r.p.m. for 1 h. The pellets were then resuspended in TNE buffer (10 mM Tris [pH 7.4], 100 mM NaCl, 1 mM ethylenediaminetetraacetic acid (EDTA), and the mixture was loaded onto a 10 to 80% sucrose gradient and centrifuged in a TH-641 rotor for 3 h at 37,000 r.p.m. The virus band was collected and pelleted in a F40L-8 × 100 rotor for 1 h at 37,000 r.p.m. The purified particles were resuspended in PBS buffer (pH 7.4) for later use.

Highly pathogenic A/Vietnam/1203/2004 (H5N1) was propagated in the allantoic cavity of embryonated chicken eggs at 37 °C for 24 h and were then aliquoted and stored at −80 °C. All experiments using live, highly pathogenic A/Vietnam/1203/2004 were reviewed and approved by the institutional biosafety program at the University of Georgia and were conducted in enhanced biosafety level 3 (BSL3+) containment according to guidelines for the use of select agents approved by the CDC.

### Construction of PIV5 AVLP plasmids

A plasmid containing PIV5 AVLP (pAVLP) was constructed from the plasmid containing the full-length genome of PIV5 (21). The target genes such as EGFP or H5 were inserted onto the pAVLP between V/P and hygromycin genes to obtain pAVLP–EGFP or pAVLP–H5. M1 of H5N1 was inserted between V/P and H5 with proper flanking gene junction sequences to generate pAVLP–M1H5. Hygromycin B and TK gene fusion (HT), or hygromycin-TK-nanoluciferase fusion (HTL) was used to replace Hyg B in pAVLP–EGFP to generate pAVLP–HT–EGFP and pAVLP–HTL–EGFP, respectively. The length of PIV5 AVLP genome was maintained as a multiple of six. The details about plasmids construction are available on request.

### Establishment of stable cell lines carrying PIV5 AVLP genome

The plasmid pAVLP–EGFP, H5, M1H5, pAVLP–HT–EGFP, or pAVLP–HTL–EGFP (3 μg), along with plasmids pCAGGS–PIV5–L (1.5 μg), pCAGGS–PIV5–NP (1 μg), pCAGGS–PIV5–P (400 ng), and pBH437 (expressing T7 polymerase, 1 μg) were mixed with 16 µl of jetPRIME transfection reagent in 500 µl of jetPRIME buffer and incubated for 10 min at room temperature. The mixture was transfected into BHK21 cells in a 100 mm dish. The Hygromycin was added at 2–4 days post-transfection at a final concentration of 250 μg/ml. The surviving cells were developed into stable BHK21 cell lines carrying PIV5 AVLP genome.

To get AVLP particles, the plasmids expressing PIV5 F, HN, and M at a ratio of 2:1:1 were transfected into BHK21–AVLP cells. The supernatants were cleared by centrifuge at 3000 r.p.m. for 10 min at 2–3 days post-transfection. To determine the titer, AVLP were serially diluted and used to infect BHK21 cells. One day after infection, the number of fluorescent cells was counted directly for AVLPs containing EGFP reporter genes or after IFA staining with anti-PIV5-P/V. The titer was determined for AVLP as amplifying particles per milliliter (AP/ml).

To sequence the PIV5 AVLP genome in the cells, the AVLP genome RNA were extracted from the cells using the RNeasy Mini Kit (QIAGEN), and reverse transcription (RT) was performed with PIV5 gene-specific primers. The reverse transcription product was further amplified by PCR using specific primers covering the whole PIV5 AVLP genome. The PCR products were sequenced.

### Indirect immunofluorescence assay (IFA)

Expressions of viral proteins and antigens in cells were examined by IFA as described before (3). Briefly, cells were fixed with 4% formaldehyde in PBS (pH 7.4) for 10 min, and then treated with 0.1% Triton X-100 plus 1% BSA, or 1% BSA in PBS for 30 min at room temperature. Fixed cells were incubated for 1 h with primary antibody at 37 °C and then incubated with FITC-conjugated secondary antibody. ProLong^®^ Gold Antifade Mountant (Life technologies) was applied directly to fluorescently-labeled cells. Fluorescence was examined and photographed using a Nikon FXA fluorescence microscope and a Nikon Eclipse Ti confocal microscope.

### Western blotting (WB)

Cells were lysed with whole-cell extraction buffer (50 mM Tris-HCl [pH 8], 280 mM NaCl, 0.5% NP-40, 0.2 mM EDTA, 2 mM, and 10% glycerol) (27). The lysates were cleared by centrifugation at 4000 r.p.m. for 15 min, and the supernatants were mixed with the same volume of 2x sodium dodecyl sulfate (SDS) loading buffer (100 mM Tris-HCl [pH 6.8], 20% glycerol, 4% SDS, 200 mM dithiothreitol [DTT], and 0.1% bromophenol blue), heated at 95 °C for 5 min, and resolved by 10% SDS polyacrylamide gel electrophoresis (SDS-PAGE). The proteins were transferred onto a polyvinylidene difluoride (PVDF) membrane using an iBlot dry blotting system (Invitrogen). The membrane was incubated with mouse anti-H5 antibody, mouse anti-PIV5–V/P, or mouse anti-PIV5–NP antibody, followed by incubation with goat anti-mouse secondary antibody labeled with horseradish peroxidase (HRP). After washing, the PVDF membrane was incubated with ECL Advance Substrate (GE Healthcare) and scanned using a Kodak Image Station 440.

### Transmission electron microscopy

Concentrated PIV5 or AVLP particles were absorbed onto Parlodion-coated nickel grids for 30 s. Grids were then floated on a drop of Tris-buffered saline (TBS), pH 7.4, for 5 min, followed by floating on drops of 3% ovalbumin in TBS for 1 h with PIV5 HN-specific mouse monoclonal antibody diluted to 1:300 in 1% ovalbumin in TBS. After washing with TBS three times, samples were incubated for 1 h with goat anti-mouse IgG coupled to 10-nm gold particles diluted at 1:10 in 1% ovalbumin in TBS. Grids were again washed with TBS and then stained with 2% phosphotungstic acid, pH 6.6. The grids were then examined using a JEOL 1230 transmission electron microscope (JEOL, Tokyo, Japan).

### Infection of AVLP particles to cells

Cells were seeded one night before infection. On the day of infection, theculture medium was removed, and AVLP diluted in DMEM/1% BSA at the indicated MOI were added and incubated for 4~6 h. We replaced the inoculum with complete cell culture medium after incubation and culture the cells for the indicated time points depending on the experiments.

### Flow cytometry of PBMCs

Human PBMCs were infected with AVLP–EGFP at an MOI of 3 or mock infected. Two days postinfection, cells were harvested and blocked with Fc Block (BD Biosciences), and stained with APC-Cy7 mouse anti-human CD3 antibody and PE-Cy7 mouse anti-human CD14 antibody (BD Biosciences). Data were then acquired with a BD LSR II flow cytometer (BD Biosciences).

### Animal studies

Six-week-old to 8-week-old, female, BALB/c mice were used in the animal studies. All animal experiments were performed following protocols approved by the Institutional Animal Care and Use Committee, University of Georgia. The mice were first anesthetized by intraperitoneal injection of tribromoethanol (Avertin; 180 to 250 μl/kg of body weight) and then inoculated intranasally with 100 μl of inoculum.

For monitoring the expression of AVLP in vivo, 2 mice per treatment were infected with PBS, AVLP–HTL–EGFP, and inactivated AVLP–HTL–EGFP (inactivated by UV irradiation at 1.3 × 10^5^ μJ/cm^2^ for 15 min on ice in a UV Stratalinker 1800 (Stratagene)). In vivo imaging was performed on anesthetized mice. Nano-Glo^®^ Luciferase Assay Substrate (Promega) was diluted 1:20 in PBS, and 100 µl was injected retro-orbitally. Bioluminescent images were acquired with IVIS Lumina XR machine, and analysis was performed using the Living Image software (PerkinElmer). Flux measurements were acquired from regions of interest automatically gated to the signal contours. All data in composite images utilized the same scale.

For testing the efficacy of AVLP–H5 as a vaccine, 15 mice per group randomly assigned were vaccinated with PBS, AVLP–H5 (4 × 10^5^ AP), AVLP–H5 (4 × 10^4^ AP), and PIV5–H5 (4 × 10^5^ PFU), respectively, on Day 0 of the study. At 21 days postinfection (DPI), 5 mice per group were euthanized, and splenocytes were processed for ELISPOT assay. At 28 DPI, the remaining mice were challenged with 10 LD_50_ of A/VN/1203/2004(H5N1). At 4 days post challenge, 5 mice per group were euthanized, and the lung was collected for measuring lung viral titer and pathological changes. The mice were weighed at least every other day and monitored twice daily for clinical signs of disease. Mice were euthanized if predetermined humane endpoints were reached. All of the surviving mice were humanely euthanized at 10 days post challenge.

For testing the efficacy of AVLP–M1H5 as a vaccine, 15 mice per group was vaccinated with PBS, AVLP–H5 (4 × 10^5^ AP), AVLP–M1H5 at a dose of 4 × 10^5^ AP, 4 × 10^4^ AP, and 4 × 10^3^ AP, respectively, on day 0 of the study. At 21 DPI, 5 mice per group were euthanized, and splenocytes were processed for ELISPOT assay. At 30 DPI, the remaining mice were challenged with 10 LD_50_ of A/VN/1203/2004(H5N1) and monitored as described above. Surviving mice were humanely euthanized at 14 days post challenge.

### Enzyme-linked immunosorbent spot (ELISPOT)

Cells were stimulated with 0.2 µg of HA or M1 antigen, or with phorbol myristate acetate-ionomycin (PMA/ION) as a positive control. The procedure followed the manual for the Mouse IFN-γ ELISPOT Kit (BD Biosciences). Spots were counted using an ImmunoSpot (Cellular Technology, Ltd.). The results are presented as mean numbers of IFN-γ -secreting cells per 10^6^ splenocytes.

### Data availability

Materials described in the manuscript, including all relevant raw data, will be freely available to any scientist wishing to use them for non-commercial purposes, without breaching participant confidentiality.

## Electronic supplementary material

Supplemental Figurres
