# Supplementary material for: Developing a platform system for gene delivery: amplifying virus-like particles (AVLP) as an influenza vaccine
Source: NPJ Vaccines. 2017 Nov 20;2:32. doi: 10.1038/s41541-017-0031-7 (PMC5696535; doi:10.1038/s41541-017-0031-7)
Supplement: Supplementary file 1 — Supplemental Figurres [file 41541_2017_31_MOESM1_ESM.docx]

**Supplement Materials**


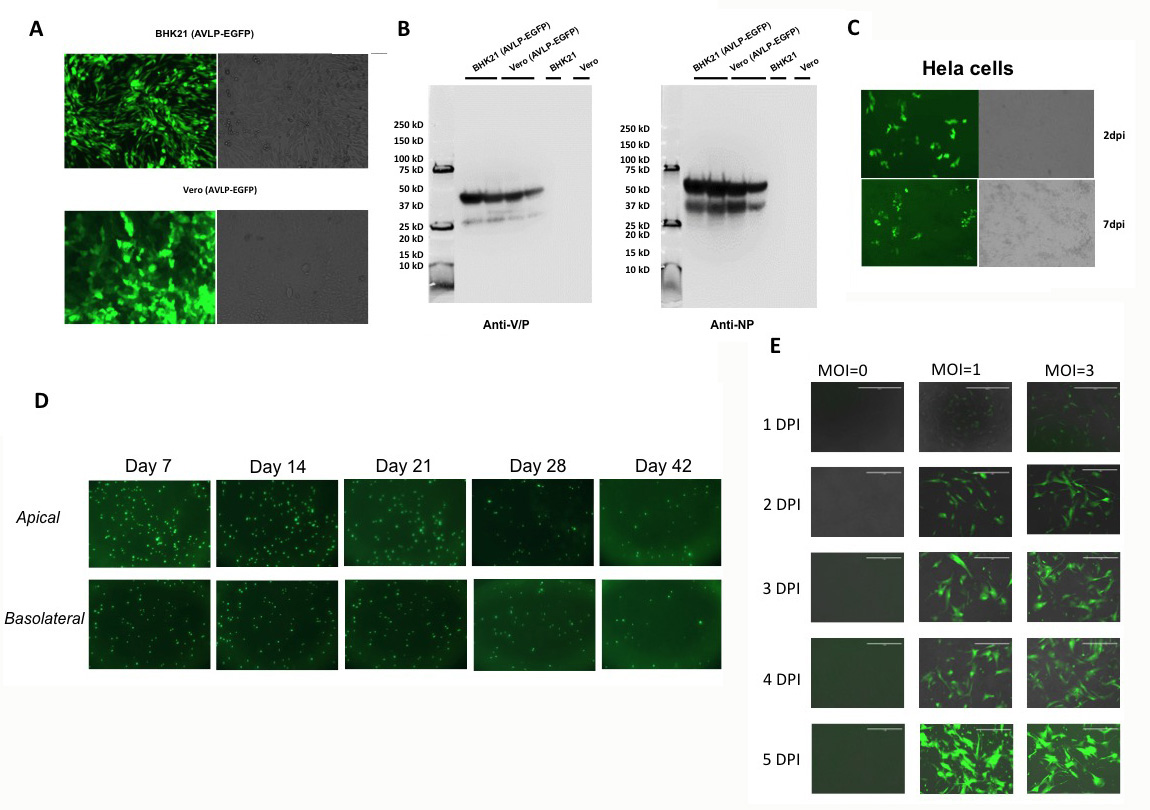


**FIG. S1 Establishing and analyzing AVLP-EGFP**. (A) Establishing cells expressing AVLP-EGPF. (B) Confirmation of expression of PIV5 proteins. Samples derived from the same experiment and gels/blots were processed in parallel. (C) HeLa cells infected with AVLP-EGPF. (D) Swine airway epithelial cells infected with AVLP-EGFP. (E) canine MSC cells infected with AVLP-EGFP.

**
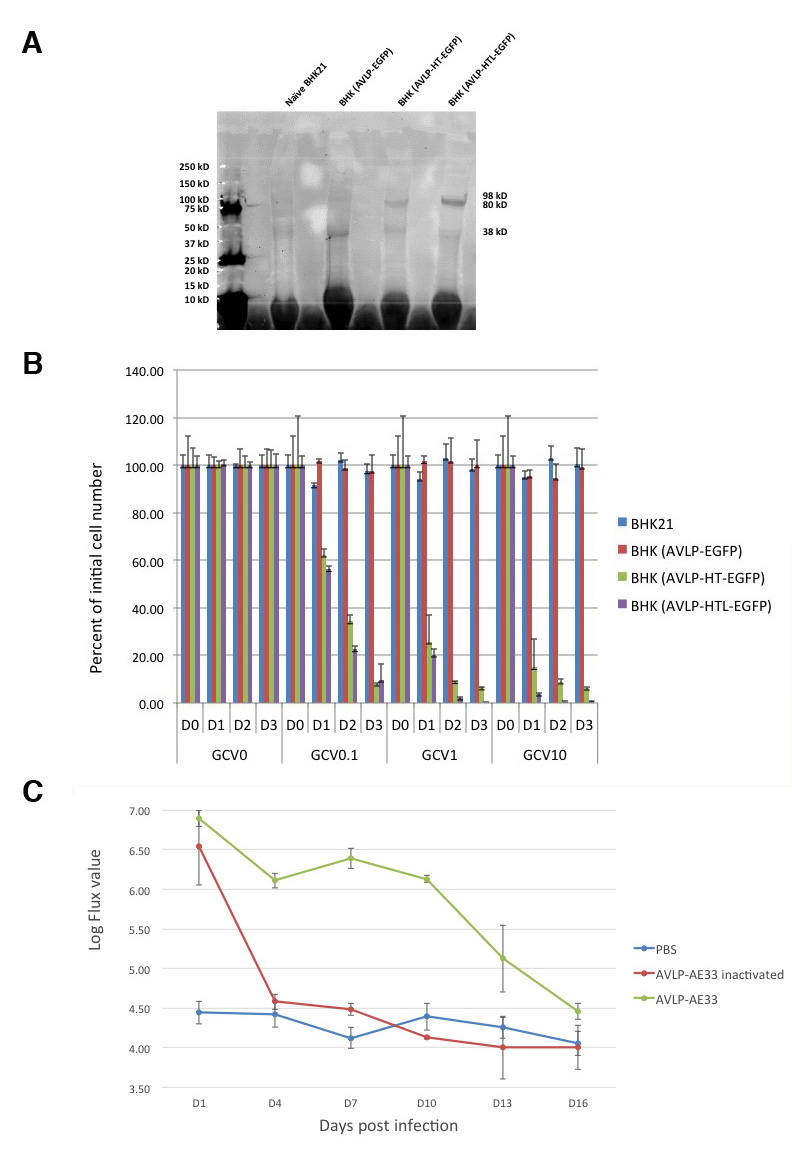
**

**FIG. S2 Analyzing AVLP-HTL-EGFP**. (A) Detection of HT or HTL fusion protein expression in BHK stable cell lines. Samples derive from the same experiment were processed in parallel. (B) Effects of GCV in cells. (C) Quantification of AVLP-based gene expression *in vivo*.


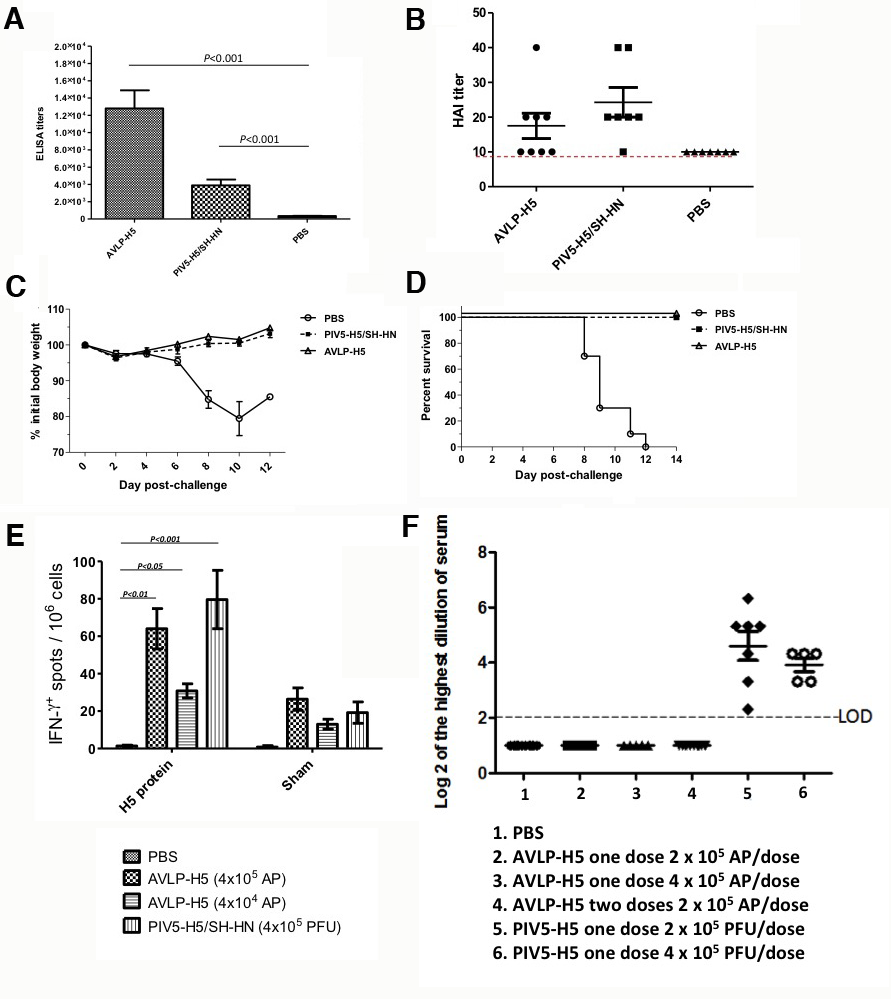


**FIG. S3 Immune responses to and protection by AVLP-H5.** (A) ELISA titers of immunized mice. BALB/c mice were intranasally immunized with AVLP-H5 (2 x 10^4^ AP) or PIV5-H5 (2 x 10^4^ PFU) and bled on day 26 post prime immunization. The mouse blood samples were collected for analysis. Purified recombinant HA protein was used to coat the ELISA plates. Titers for each mouse were determined at the highest dilution that showed an OD_450_ value above the mean ± 2 SD of the serum from the PBS control group. (B) HAI titers of anti-H5 antibodies in mice. 4 HAU of the influenza A virus (A/Vietnam/1203/04) were mixed with serially-diluted mouse sera in 96-well round-bottom plates. The hemagglutination inhibition (HAI) titer was scored as the reciprocal of the highest dilution antiserum that completely inhibits hemagglutination. The graph shows the mean value of duplicate wells for each mouse. The limit of detection of the HAI titer (10) is indicated by a dashed line. (C and D) AVLP-H5 protection against H5N1 virus challenge. Mice were vaccinated intranasally with a single dose of PBS or PIV5-H5 (n = 10, 2x10^4^ PFU), or two doses of AVLP-H5 19 days apart (n = 15 per group, 2x10^4^ AP each). At day 42 post prime vaccination, mice were challenged with 10 LD_50_ of H5N1 influenza A virus (A/Vietnam/1203/04). Weight loss (C) and survival (D) were monitored at two day intervals for 14 days following influenza virus challenge. Weight loss is graphed as an average percentage of the original weight (the day of challenge). (E) Elispot to analyze T cell responses. Five mice per group were infected intranasally with PBS, AVLP-H5 (4 x 10^5^ AP/ml), AVLP-H5 (4 x 10^4^ AP/ml), and PIV5-H5 (4 x 10^5^ PFU/ml). At 21 DPI, mice were euthanized, and splenocytes were processed for ELISPOT assay. (F) Neutralizing antibody titers in mice immunized with AVLP-H5. BHK21 cells were seeded into 96-well plates one night before infection. Mice serum samples were treated at 56°C for 30 min, serially diluted with DMEM with 1% BSA and mixed at equal volume with 200 TCID_50_ of PIV5 wild type virus. After being incubated at 37°C for 1 h, serum-virus mixture was added onto BHK21 cells in 96 well plate. PIV5-specific cytopathic effect (CPE, syncytia) was observed after 3 days culture at 37°C incubator with 5% CO_2_. Triplicate was performed for each serum sample. The titer of each sample was recorded as the highest dilution that did not show any CPE.


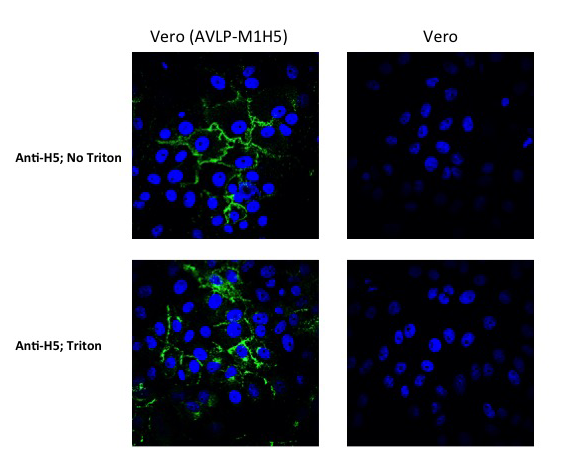


**FIG. S4 Expression of H5 protein in AVLP-M1H5-containing Vero cells.** Cells were infected with AVLP-M1H5 and then treated with or without triton in an IFA assay.


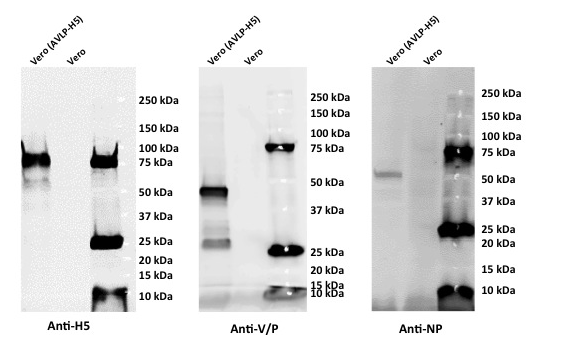


**FIG. S5 Detection of H5, PIV5 V/P, and NP expression in cells by western blotting.** The Vero (AVLP-H5) cell samples were stained with anti-H5, anti-PIV5 V/P, or anti-PIV5 NP antibodies. Vero cell samples were used as a negative control. Samples derived from the same experiment and gels/blots were processed in parallel. This is the same figure as Figure 4B.


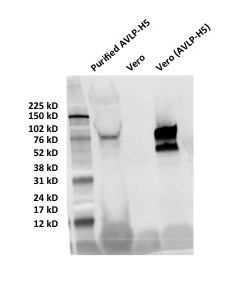


**FIG. S6 H5 incorporation.** The purified AVLP-H5 particles were subjected to WB analysis with H5 specific antibody. Samples derived from the same experiment and gels/blots were processed in parallel. HW created this figure. This is the same figure as Figure 4E.


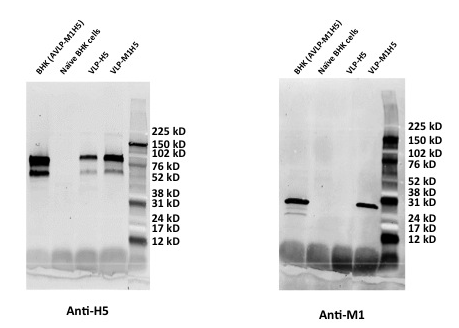


**FIG. S7 Detection of M1 and H5 in cell media.** Supernatants from BHK (AVLP-H5) and BHK (AVLP-M1H5) cell cultures were purified and subjected to WB. Samples derived from the same experiment and gels/blots were processed in parallel. This is the same figure as Figure 6A.


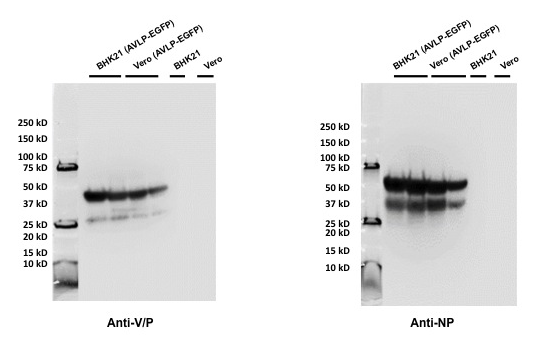


**FIG. S8 Confirmation of expression of PIV5 proteins.** Samples derived from the same experiment and gels/blots were processed in parallel. This is the same figure as Figure S1B.


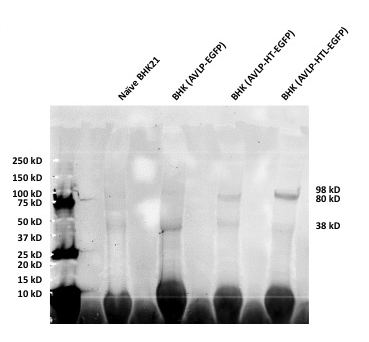


**FIG. S9 Detection of HT or HTL fusion protein expression in BHK stable cell lines.** This is the same figure as Figure S2A.
